# Public Awareness and Knowledge of Prostate Cancer Screening: A Community Study in Saudi Arabia

**DOI:** 10.3390/healthcare13161962

**Published:** 2025-08-11

**Authors:** Geetha Kandasamy, Khalid Orayj, Yahya I. Asiri, Eman Shorog, Asma M. Alshahrani, Hebah Abdullah Alenazi

**Affiliations:** 1 Department of Clinical Pharmacy, College of Pharmacy, King Khalid University, Abha 62529, Saudi Arabia; 2 Department of Pharmacology, College of Pharmacy, King Khalid University, Abha 62529, Saudi Arabia; 3 Department of Clinical Pharmacy, College of Pharmacy, Shaqra University, Dawadimi 11961, Saudi Arabia; 4 Innova Healthcare, Riyadh 13214, Saudi Arabia

**Keywords:** prostate cancer, knowledge, awareness, barriers, screening tests

## Abstract

**Background**: Prostate cancer (PCa) is one of the most common malignancies among men in Saudi Arabia and contributes significantly to cancer-related morbidity and mortality. The objective of this survey was to evaluate community awareness and screening practices related to PCa among men in the Asir region of Saudi Arabia. **Method**: A cross-sectional study was conducted from 5 October to 25 December 2024 among men aged 40 and above in the Asir region, excluding those with a prior PCa diagnosis. Using convenience sampling, 399 participants were recruited via social media and community outreach. Data were collected through a self-administered online questionnaire covering demographics, medical history, PCa knowledge, information sources, prevention, screening awareness, and barriers. Informed consent was obtained from all participants. **Results**: The study comprised 399 male participants, with 37.09% aged 40–50, 36.34% aged 51–60, and 26.56% over 60. Most participants (363; 90.97%) were married, 245 (61.4%) had a university education, 282 (70.67%) lived in urban areas, and 180 (45.11%) were employed. Over half of the participants, 222 (55.63%), had a personal history of prostate problems. Additionally, 272 (68.17%) had health insurance, and 153 (38.34%) reported a monthly income between 10,000 and 14,999 SAR. The study found that 329 (82.5%) participants had good knowledge of PCa but only 197 (49.4%) had good awareness of screening methods. Key predictors of good awareness of PCa screening included a personal history of prostate problems (odds ratio—OR = 4.791, *p* = 0.000, confidence interval—CI 2.727–8.418) and health insurance (OR = 0.359, *p* = 0.000, CI 0.203–0.636). Common barriers to screening were affordability, *n* = 116 (29.07%), and perceived good health, *n* = 201 (50.37%). Additionally, 154 participants (38.59%) found screening uncomfortable, while 156 (39.59%) believed the Digital Rectal Exam (DRE) was harmful or embarrassing. Significant differences in perceived barriers were found based on age (F = 11.449, *p* < 0.001), education (F = 2.608, *p* = 0.051), occupation (F = 3.668, *p* = 0.026), family history (F = 17.407, *p* < 0.001), and income (F = 5.148, *p* = 0.006). **Conclusions**: The study highlights a significant gap between general knowledge and specific awareness of prostate cancer (PCa) screening among men in the Asir region. Although 82.5% demonstrated good overall knowledge, only 49.4% were aware of screening methods, and just 44.36% had undergone PSA testing. Common barriers included perceived good health, fear of diagnosis, embarrassment, and financial concerns. However, due to the use of convenience sampling, online distribution, and geographic restriction to the Asir region, the findings may not be generalizable to the broader male population in Saudi Arabia, particularly older men and those in rural areas. Addressing these gaps requires targeted education, empowerment of healthcare providers, and coordinated public health strategies to enhance early detection and reduce the PCa burden.

## 1. Introduction

Prostate cancer (PCa) is a significant global health issue and one of the most frequently diagnosed cancers in men, with approximately 1.3 million new cases reported worldwide in 2018 [1,2]. The incidence is notably higher in developed countries, largely due to the widespread use of prostate-specific antigen (PSA) testing [3]. The global burden is projected to rise to 1.7 million new cases and 500,000 deaths by 2030 [4]. In the Arab world, both incidence and mortality are increasing, with more patients presenting at advanced stages compared to Western countries like the United States [5,6].

In Saudi Arabia, PCa is the second most common malignancy among men over 60 years, with an age-standardized incidence rate of 5.1 per 100,000 males recorded between 2001 and 2008 [7]. Despite this, awareness and screening remain suboptimal. International guidelines recommend early detection through PSA testing and digital rectal examination (DRE), especially in high-risk groups such as men with a family history or germline mutations. For instance, the American Urological Association suggests initiating screening between the ages of 40 and 45 for high-risk individuals [8,9]. Early detection, particularly of low-grade PCa, has been shown to significantly improve prognosis and survival [10]. The European Randomized Study of Screening for Prostate Cancer (ERSPC) demonstrated reduced mortality through PSA-based screening [11], while the American Cancer Society underscores the need for informed screening decisions [12].

The national cancer registry data ranked PCa as the fourth most frequent cancer among Saudi men in 2014, with 323 new cases and an incidence rate of 5.5 per 100,000. Higher rates were observed in the Eastern Province (12.6) and Riyadh (9.5), with an average diagnosis age of 73 years [13]. A national review also reported an eightfold increase in incidence from 1990 to 2016 [14]. In the Asir region specifically, Otifi et al. found an 8.7% prevalence among 883 patients between 2008 and 2018 [15]. Despite rising trends, PCa remains underdiagnosed due to limited awareness of risk factors, symptoms, and available screening options. This gap can result in delayed diagnosis and poorer health outcomes. Assessing knowledge and behaviors around PCa screening is thus essential to guide public health efforts.

The Asir region in southwestern Saudi Arabia, home to over two million people, comprises both urban and rural populations across challenging mountainous terrain [16]. Although national campaigns by the Saudi Ministry of Health aim to improve cancer awareness and screening, disparities persist, particularly in peripheral and rural communities. Cultural beliefs, limited access to specialist care, and fewer screening facilities may contribute to low awareness and late detection in this region.

Notably, the Asir region remains under-represented in public health research on PCa. Understanding community perceptions and practices is critical for identifying knowledge gaps and developing targeted education strategies. Therefore, this study aims to evaluate awareness, knowledge, and screening behaviors related to prostate cancer among men in the Asir region of Saudi Arabia.

## 2. Method

### 2.1. Study Design/Inclusion and Exclusion Criteria

A cross-sectional study was conducted from 5 October to 25 December 2024 among men aged 40 and above residing in the Asir region of Saudi Arabia. Those with a prior diagnosis of prostate cancer or who declined to provide informed consent were excluded.

### 2.2. Sampling Procedure and Sample Size

Study participants were selected using a non-probability snowball sampling method, which is particularly effective for reaching dispersed or hard-to-access populations. To ensure diversity in participant characteristics, the snowball sampling strategy began with a carefully selected group of initial participants (“seeds”) who represented a range of demographic profiles, including variations in age, educational background, occupation, and geographic location within the Asir region. These initial participants were encouraged to disseminate the survey within their own social and professional circles. This approach was designed to promote wider outreach and facilitate the inclusion of individuals from both urban and rural areas, enhancing the demographic representativeness of the sample.

**Figure 1:**
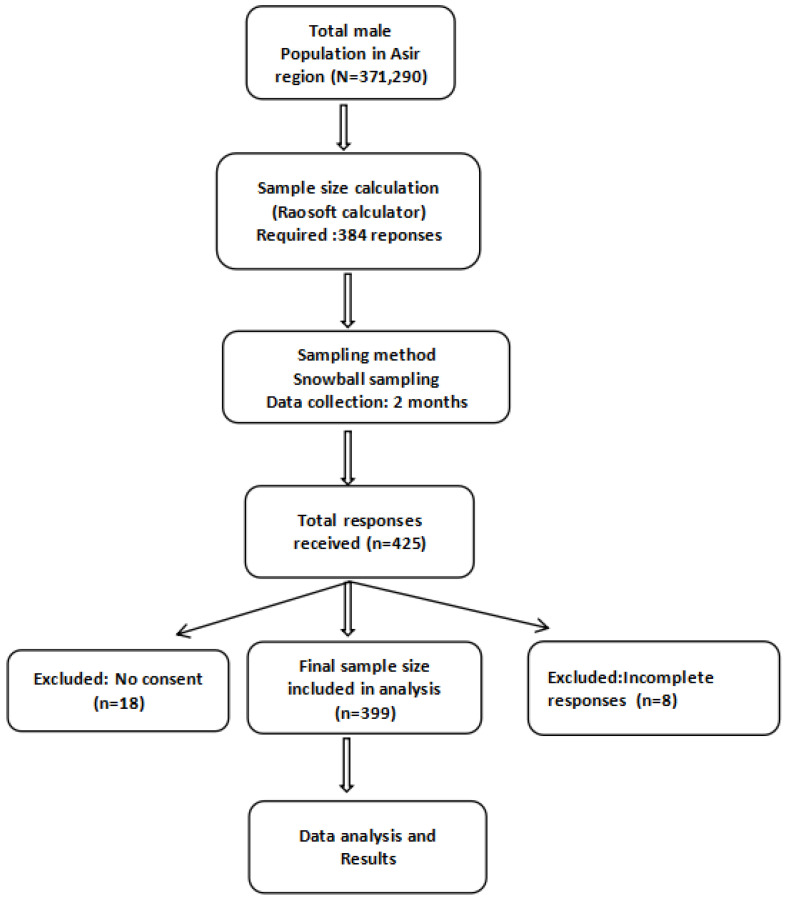
Flow chart illustrating the sampling procedure and participant inclusion process.

Prior to the commencement of the study, the total male population of the Asir region (371,290) was considered to calculate the required sample size. The calculation was performed using the Raosoft sample size calculator [17], which is based on Cochran’s formula, applying standard parameters for cross-sectional surveys: a 95% confidence level, 5% margin of error, and an anticipated response distribution of 50%. This 50% prevalence was chosen as a conservative estimate to maximize sample size in the absence of prior data on prostate cancer screening awareness. Based on these parameters, the minimum estimated sample size was 384 participants. A total of 425 responses were received; however, 26 were excluded: 8 due to incomplete forms and 18 due to lack of consent. Therefore, 399 participants were included in the final analysis, exceeding the minimum required sample size (Figure 1).

### 2.3. Participant Recruitment Process

Initial participants were identified through personal networks, including friends, colleagues, and members of community organizations focused on men’s health. The questionnaire was prepared using Google Forms, and the survey link was then shared via various social media platforms, including Twitter and WhatsApp, as well as through community care centers. To promote inclusivity and broaden participation, the survey was made available in Arabic, the native language of the majority of the population in Saudi Arabia, and was disseminated through community leaders and healthcare professionals across the Asir region. This collaborative approach enabled outreach to individuals from diverse demographic backgrounds varying in age, education level, and socioeconomic status, thereby enhancing the representativeness of the sample. Written informed consent was obtained from all participants before data collection commenced. An informed consent form, detailing the study’s objectives, was provided at the beginning of the questionnaire. Participants who consented were then directed to proceed to the next section. They were assured that participation was voluntary and anonymous and that all personal information would remain confidential and be processed anonymously.

### 2.4. Data Collection Tool and Questionnaire Validation

A self-administered, structured questionnaire consisting of four main sections was used to collect data. The questionnaire was adapted from previously published research studies [18,19,20]. Initially written in English, it was subsequently translated into Arabic, the native language of the majority of the population in Saudi Arabia. A native Arabic speaker with subject-matter expertise conducted the translation to ensure both linguistic accuracy and cultural relevance.

Prior to the main study, a pilot study involving 50 men from the target population was conducted to evaluate the clarity, language, cultural relevance, and comprehensibility of the questionnaire. Feedback obtained from this pilot testing was instrumental in refining the questionnaire to ensure that all questions and instructions were clearly understood and consistently interpreted by the participants. Participants involved in the pilot study reported that the questionnaire was easy to understand, with no significant ambiguities or misinterpretations, confirming its appropriateness for the target population. This process enhanced the overall robustness and validity of the data collection tool. Additionally, a panel of experts reviewed the format and content of the questionnaire to assess its overall content validity. The reliability of the questionnaire was assessed using Cronbach’s alpha, which yielded values of 0.72 for the knowledge section, 0.83 for awareness, and 0.84 for barriers, indicating satisfactory to good internal consistency.

### 2.5. Questionnaire Description

The survey questionnaire gathered information on socioeconomic status, personal and family medical history of PCa, knowledge of PCa, sources of information, prevention strategies, awareness of PCa screening techniques, and barriers to screening.

Part 1: The socioeconomic portion covered the following topics: age, marital status, education level, place of residence, employment, history of prostate problems, family history of prostate problems, health insurance status, and monthly family income.

Part 2: Respondents’ knowledge was evaluated through seven questions covering the risk factors around, symptoms of, and prevention of PCa.

Part 3: Awareness of PCa screening methods includes eight statements, such as the various examinations available for cancer detection, the fact that early screening can enhance survival rates, personal experiences with PSA tests, and the frequency of such tests. It also addressed whether only men with urinary symptoms should be screened, the significance of regular prostate examinations, and whether individuals have performed these examinations themselves.

Part 4: Barriers to prostate cancer (PCa) screening were assessed using a 4-point Likert scale across 10 items. Each item represented a potential barrier, including the inability to afford screening, the belief that one is healthy, uncertainty about where to go for screening, fear of discovering a health problem, the perception that PCa screening is time-consuming, concerns about discomfort during the procedure, embarrassment or fear related to the digital rectal exam (DRE), fear of potential impotence, the belief that nothing can prevent PCa, and the belief that PCa is not a serious disease. Responses were rated as 1 = strongly disagree, 2 = disagree, 3 = agree, and 4 = strongly agree. The individual scores from each item were summed to generate a total barrier score for each participant, with higher scores indicating a greater perceived level of barriers to screening.

### 2.6. Scorings

Participants received a score of 1 for each correct answer and 0 for each incorrect answer. Operational Definition—Knowledge of Prostate Cancer: A total of 14 points was possible in the knowledge section. Participants with scores ≥ 7 were classified as having “good” knowledge, while those with scores < 7 were classified as having “poor” knowledge. Awareness of Prostate Cancer: The awareness section, which consisted of a maximum of 8 points, categorized participants with scores ≥ 4 as having “good” awareness, while those with scores < 4 were classified as having “poor” awareness [20,21].

### 2.7. Ethical Considerations

The study’s ethical framework was approved by the Institutional Review Committee at King Khalid University (ECM # 2024-3111). All methods were performed in accordance with the relevant guidelines and regulations. Written consent was obtained from all participants before data collection commenced.

### 2.8. Data Analysis

Statistical analysis was performed using SPSS Statistics, version 20.0 for Windows. Frequencies and percentages were used to describe the socioeconomic characteristics of the study participants. Multivariate logistic regression analysis assessed significant associations between independent variables (socioeconomic groups) and dependent variables (knowledge and awareness). Additionally, *t*-tests and ANOVA were conducted to identify differences in barriers across socioeconomic groups, with the significance level set at *p* < 0.05.

## 3. Results

A total of 399 male participants were included in the study. As shown in Table 1, the majority were aged 40–60 years, *n* = 294 (73.4%), and married, 363 (91%). Most participants had a university degree, *n* = 245 (61.4%), and lived in urban areas, *n* = 282 (70.7%). A personal history of prostate problems was reported by 222 participants (55.6%) and a paternal family history by 184 (46.1%). Health insurance coverage was reported by 272 participants (68.2%). The largest income group earned 10,000–14,999 SAR, *n* = 153 (38.3%). A total of 180 participants (45.1%) were employed (Table 1).

As shown in Table 2, good knowledge of prostate cancer (PCa) was found in 329 participants (82.5%), whereas good awareness of screening was present in only 197 (49.4%). The mean knowledge score was 9.47 (median = 10.00; range = 14.00), whereas the mean screening awareness score was 4.57 (median = 4.00; range = 8.00), suggesting that while most participants had a relatively high level of knowledge, their awareness regarding screening methods was comparatively lower. As shown in Table 3, most participants knew what PCa is, *n* = 335 (83.95%), and believed it to be preventable, *n* = 300 (75.18%). Key identified risk factors included age over 50, *n* = 336 (84.21%); family history, *n* = 287 (71.92%); poor physical activity, *n* = 272 (68.17%); and smoking, *n* = 247 (61.9%). Frequently reported symptoms included blood in urine, *n* = 299 (74.93%), erectile dysfunction, *n* = 295 (73.93%), and lower back pain, *n* = 280 (70.17%). The most commonly selected age range for PCa screening was 41–50 years, *n* = 203 (50.87%). Primary sources of information were healthcare providers, *n* = 146 (36.59%), mass media, *n* = 112 (28.07%), and the internet, *n* = 98 (24.56%).

Table 4 (logistic regression analysis of variables associated with knowledge of prostate cancer among study participants) presents the association between various factors and participants’ knowledge levels. The analysis revealed that participants with a secondary level of education had significantly lower odds of having good knowledge about prostate cancer compared to those with a university-level education (odds ratio [OR] = 0.460, 95% confidence interval [CI]: 0.227–0.933, *p* = 0.031).

Due to the group size imbalance between secondary and university education levels (*n* = 81 vs. *n* = 245) and potential confounding, two models were analyzed: an unadjusted model with education only, and an adjusted model including occupation, income, residence, and health insurance. In the unadjusted model (Table 5), secondary education was significantly associated with lower knowledge (OR = 0.476, 95% CI: 0.258–0.878; *p* = 0.018). This association remained significant in the adjusted model (Table 6; OR = 0.481; 95% CI: 0.242–0.957; *p* = 0.037), indicating minimal confounding. While unemployment was also significant (OR = 2.574; 95% CI: 1.084–6.110; *p* = 0.032), it did not affect the education effect. These results support the robustness of the original model and confirm that secondary education independently predicts lower knowledge of prostate cancer, with no need to alter the model based on moderate group size differences.

Table 7 summarizes key aspects of awareness and uptake of prostate cancer (PCa) screening. A total of 29.3% were unaware of any screening methods, while 76.9% believed early screening improves survival. PSA testing had been undertaken by 44.4%, though 36.3% could not recall. Treatment awareness was limited, with only 29.8% knowing about drugs or chemotherapy.

Table 8 presents logistic regression results identifying significant predictors of PSA test uptake. Unmarried individuals were significantly less likely to undergo testing (OR = 0.274; 95% CI: 0.084–0.898; *p* = 0.033). Participants with a personal history of prostate problems were over four times more likely to have undergone testing (OR = 4.107; 95% CI: 2.296–7.348; *p* < 0.001). Lack of health insurance was associated with lower testing rates (OR = 0.428; 95% CI: 0.232–0.790; *p* = 0.007). A family history of prostate problems through maternal relatives (OR = 4.033; 95% CI: 1.676–9.707; *p* = 0.002) and fathers (OR = 1.758; 95% CI: 1.017–3.040; *p* = 0.043) significantly increased the likelihood of testing. Other demographic variables were not statistically significant.

Table 9 presents the logistic regression analysis of variables associated with awareness of prostate cancer screening among the study participants. Logistic regression analysis showed that a personal history of prostate problems was strongly associated with good awareness (OR = 4.791, 95% CI: 2.727–8.418, *p* < 0.001). Having health insurance also significantly predicted good awareness (OR = 2.786, 95% CI: 1.573–4.935, *p* < 0.001). Participants with health insurance were nearly 2.8 times more likely to have good awareness compared to those without insurance.

Table 10 indicates that among men without a personal history of prostate problems, awareness of prostate cancer screening was significantly associated with health insurance, occupation, and family history. Participants with health insurance were over twice as likely to be aware of screening (OR = 2.288, 95% CI: 1.025–5.109, *p* = 0.043), while unemployed individuals had higher awareness compared to retirees (OR = 5.834, 95% CI: 1.332–25.552, *p* = 0.019). Additionally, those with a paternal family history of prostate problems were more likely to be aware than those with no known history (OR = 2.488, 95% CI: 1.059–5.847, *p* = 0.037).

Table 11 highlights key barriers to prostate cancer (PCa) screening, revealing that psychological, informational, and financial factors significantly hinder participation. The most commonly cited obstacles were a perceived lack of need due to good health (69.4%), fear of diagnosis (67.7%), and uncertainty about where to go for screening (65.9%). Other notable concerns included the potential negative impact on sexual health (60.4%), discomfort with the procedure (59.4%), embarrassment or harm associated with DRE (58.9%), and financial constraints (57.4%). These findings underscore the need for targeted interventions that address emotional concerns, improve knowledge of screening services, and mitigate cost-related barriers.

Table 12 presents the results of *t*-tests and ANOVA analyses examining differences in perceived barriers to prostate cancer screening across various demographic and health-related variables. The independent *t*-tests showed no significant differences in barrier scores based on marital status or residence. However, participants with a personal history of prostate problems reported significantly higher barrier scores (mean = 28.26, SD = 5.94) than those without such a history (mean = 24.79, SD = 5.48; t = 6.011, *p* < 0.001). Similarly, participants with health insurance had higher barrier scores (mean = 27.71, SD = 5.98) compared to uninsured individuals (mean = 24.59, SD = 5.43; t = 4.998, *p* < 0.001). A significant difference in barrier scores was observed based on whether participants had undergone PSA testing. Those who had taken the test reported higher barrier scores (M = 28.57, SD = 6.07) than those who had not (M = 25.24, SD = 5.50), with a statistically significant difference (t = 5.733, *p* < 0.001).

ANOVA revealed significant differences in perceived barriers by age (F = 11.449, *p* < 0.001; highest in 51–60 years), occupation (F = 3.668, *p* = 0.026; higher in unemployed), family history (F = 17.407, *p* < 0.001; highest with maternal history), and income (F = 5.148, *p* = 0.006; higher in lower-income groups). Education showed a marginal effect (F = 2.608, *p* = 0.051), suggesting a possible influence on perceived barriers.

## 4. Discussion

This study assessed awareness and knowledge of prostate cancer (PCa) and its screening among men in the Asir region of Saudi Arabia. While 82.5% of participants reported general awareness of PCa, only 49.4% demonstrated knowledge of appropriate screening methods. This highlights a critical knowledge behavior gap, where awareness does not consistently translate into preventive action, particularly regarding screening methods such as DRE and PSA testing. Compared to previous studies from Jazan, Medina, Jeddah, and Makkah, which reported lower general awareness and limited knowledge of screening modalities [7,22], our findings reflect a relatively higher level of awareness but a similar shortfall in actionable knowledge. For instance, awareness of DRE and PSA testing was only 37.34% and 18.54%, respectively, comparable to findings from Kenya and Tanzania [23,24], and markedly lower than studies reporting awareness levels as high as 90% [25]. This underscores a broader trend across various regions: awareness of PCa exists, but understanding of screening tools remains insufficient, calling for targeted health education initiatives.

Our study adds novel insights by focusing on this knowledge–practice disconnect in a community-based sample from the Asir region, an area previously under-represented in PCa research. Unlike many studies that focus solely on general awareness, our findings provide actionable information relevant for public health planning and screening interventions. Awareness of PCa risk factors such as family history, advanced age, smoking, and physical inactivity was moderate and consistent with findings from Riyadh and international studies [25,26,27,28,29,30]. However, regional disparities persist. For example, although 50% of participants in our study recognized advanced age as a risk factor, lower recognition has been reported in South Africa [24], suggesting that cultural factors and regional health messaging play an important role in shaping public knowledge. Sociodemographic analysis revealed no significant association between income and PCa awareness, consistent with other Saudi studies [31]. This could reflect the impact of Saudi Arabia’s government-sponsored healthcare programs, which may help equalize access to health information. Instead, variables such as educational level, age, and employment status were more predictive of awareness, aligning with global evidence [32,33]. These findings reinforce the importance of tailoring educational interventions to less-educated populations.

Notably, health insurance coverage emerged as a strong predictor of PCa awareness; insured participants were 2.8 times more likely to possess good knowledge. This supports previous findings linking insurance status with better health literacy and screening uptake [19,34,35]. Integrating public health education into insurance-related services could therefore serve as a strategic avenue to improve screening behaviors. Healthcare providers were the most frequently reported source of information (36.59%), followed by media. This pattern contrasts with studies where media dominated [20] and aligns more closely with findings from Rwanda and Ethiopia that emphasize the role of healthcare professionals [19,36]. Given that most participants expressed belief in the benefits of early detection, consistent with earlier studies linking delayed presentation to poor outcomes [37,38], the proactive engagement of healthcare providers is crucial in enhancing PCa education and encouraging timely screening.

Despite the relatively high general awareness, screening uptake remains low, a finding echoed in studies from the U.S., Nigeria, and Cameroon, where knowledge did not reliably lead to preventive behavior [39]. For instance, Ernest et al. [40] found that only 8.1% of respondents underwent screening despite high awareness of symptoms. Similarly, Shaqran et al. [18] reported poor screening behaviors, consistent with our findings. Nevertheless, the awareness levels observed in our study were higher than those reported in Jordan and Egypt [41], suggesting geographic variability in public health education and healthcare access. Participants identified multiple barriers to screening: a perceived lack of need due to good health, fear of diagnosis, embarrassment about DRE, financial concerns, and unawareness of screening locations. These align with barriers documented in sub-Saharan Africa and Turkey, including mistrust, negligence, and logistical issues [20,42,43,44]. Interestingly, we found that perceived barriers were higher among individuals with insurance and a history of prostate issues, possibly reflecting increased awareness without adequate guidance or support to act on it.

To enhance screening uptake, comprehensive and multifaceted strategies are required. Public education campaigns should specifically target middle-aged men, especially those who are asymptomatic to challenge misconceptions and reinforce the value of early detection. Addressing psychological and logistical barriers through mobile clinics, subsidized testing, and clearer access pathways is also critical. Aligning these efforts with international screening guidelines, such as those from the American Cancer Society and the U.S. Preventive Services Task Force [45,46,47], can further bolster national prevention efforts.

It is important to acknowledge that the use of convenience sampling and online distribution may have introduced selection bias. Our sample likely included younger, more educated, and internet-literate individuals residing in urban areas of the Asir region, potentially under-representing older adults, individuals with lower educational attainment, and rural residents. This limitation may have inflated the reported awareness and knowledge levels [48]. Therefore, these findings should be interpreted as representative of the digitally accessible population in Asir rather than the broader Saudi male population. Future research using randomized or stratified sampling across diverse regions will be essential for enhancing generalizability and informing national screening policies.

### Limitations

This study has several important limitations that should be acknowledged. First, it employed a non-probability convenience sampling method, with participants recruited primarily via personal networks and social media platforms. This approach likely introduced self-selection bias, potentially over-representing younger, more educated, and health-conscious individuals, while under-representing older, rural, or less-connected populations. Due to the anonymous nature of online recruitment, we were unable to verify whether all participants were indeed adult males residing in the Asir region. Given that the study was limited to a single region and relied on an internet-active cohort, the findings may not be generalizable to the broader Saudi male population. The use of a self-administered, self-reported questionnaire also introduces the possibility of recall and social desirability biases, which may have affected the accuracy of responses. Additionally, the cross-sectional design of the study prevents the establishment of causal relationships. Another key limitation is that the study did not assess actual screening behavior, nor did it apply a behavioral framework to evaluate whether awareness translated into practice. Although we identified predictors of awareness, we did not explore whether this knowledge led to participation in screening activities. To enhance transparency and interpretation, we recommend that the abstract and conclusion clearly state that these findings primarily reflect the awareness levels of the younger, internet-active male population in the Asir region. If feasible, future analyses should examine subgroup differences—for example, comparing awareness between age groups—to illustrate how the sampling method may have influenced the results. Future studies should adopt more representative, randomized sampling strategies, include participants from both urban and rural areas, and expand data collection across multiple regions in Saudi Arabia. Incorporating validated behavioral models would also help elucidate the relationship between awareness and actual screening uptake. Until such data are available, caution is warranted in generalizing these findings to the broader population.

## 5. Conclusions

This study highlights a significant gap between general awareness and specific knowledge of prostate cancer (PCa) screening methods among men in the Asir region of Saudi Arabia. Although 82.5% of participants demonstrated good overall knowledge, less than half (49.4%) were aware of key screening tools such as the prostate-specific antigen (PSA) test and digital rectal examination (DRE), and only 44.36% had undergone PSA testing. Multivariable analysis identified statistically significant associations between screening awareness and factors such as health insurance status, marital status, and personal or family history of prostate issues. However, due to the study’s cross-sectional nature, no causal relationships can be inferred. Participants also reported several perceived barriers to screening, including good self-reported health, fear of diagnosis, embarrassment, discomfort, and financial concerns.

These findings should be interpreted with caution due to several limitations. The study employed a convenience sampling strategy and online survey format, with data collected only from the Asir region. This may limit the generalizability of results, particularly to older adults, rural populations, and individuals without internet access. Furthermore, the cross-sectional design precludes the establishment of temporal or causal relationships. To address these gaps, health authorities should implement targeted strategies to enhance screening accessibility and public awareness. This includes deploying mobile screening units in underserved areas, integrating PCa screening into primary care settings, and offering subsidized or free PSA testing for low-income and uninsured individuals. Awareness campaigns should be culturally tailored and delivered through mass media, mosques, workplaces, and community centers to reach older and less-educated men. Healthcare providers should be empowered to play a more proactive role in patient education, consistent with national and international screening guidelines. Future efforts should focus on inclusive and culturally tailored educational programs that promote informed decision-making regarding PCa screening in accordance with clinical guidelines. In addition, policymakers should prioritize eliminating psychosocial and structural barriers, such as stigma, fear, and cost, that hinder screening participation. Further research using regionally representative, randomized sampling is needed to better understand prostate cancer awareness and screening behaviors across diverse populations in Saudi Arabia.

## Figures and Tables

**Table 1:** Distribution of demographic, health, and socioeconomic factors among study participants.

Variables	Category	Number (*n* = 399) (%)
Age	40–50	148 (37.09)
	51–60	145 (36.34)
	Above 60 years	106 (26.56)
Marital status	Yes	363 (90.97)
	No	36 (9.02)
Level of education	Primary level	12 (3)
	Secondary level	81(20.3)
	University level	245 (61.4)
	Other (uneducated)	61(15.28)
Residence	Rural	117 (29.32)
	City	282 (70.67)
Occupation	Employed	180 (45.11)
	Unemployed	122 (30.57)
	Retired	97 (24.31)
Personal history of prostate problems	Yes	222 (55.63)
	No	177 (44.36)
Family history of prostate problems	Mother’s relative	40 (10)
	From father	184 (46.11)
	Not known	175 (43.85)
Health insurance	Yes	272 (68.17)
	No	127 (31.82)
Monthly family income	5000 to 9999 SAR	130 (32.58)
	10,000 to 14,999 SAR	153 (38.34)
	Above 15,000 SAR	116 (29.07)

**Table 2:** Descriptive statistics and categorization of knowledge and awareness scores related to prostate cancer screening among the study population.

Scores	Knowledge (%) (*n* = 399)	Awareness of Screening (*n* = 399) (%)
Good	329 (82.5)	197 (49.4)
Poor	70 (17.5)	202 (50.6)
Mean	9.47	4.57
Median	10.00	4.00
Range	14.00	8.00

**Table 3:** Knowledge and sources of information regarding prostate cancer among study participants.

Knowledge Questions	Yes (%)	No (%)
Do you know what prostate cancer is?	335 (83.95)	64 (16.04)
Is it possible to prevent prostate cancer?	300 (75.18)	99 (24.81)
Have you ever heard about prostate-specific antigen (PSA)?	210 (52.63)	189 (47.36)
**Prostate cancer risk factors**		
Increased age > 50 years	336 (84.21)	63 (15.78)
Family history	287 (71.92)	112 (28.07)
Poor physical activity	272 (68.17)	127 (31.82)
Smoking	247 (61.9)	152 (38.09)
**Prostate cancer symptoms**		
Difficulty in urine emission	241 (60.4)	158 (39.59)
Erectile dysfunction	295 (73.93)	104 (26.06)
Lower back pain	280 (70.17)	119 (29.82)
Lower pelvic pain	262 (65.66)	137 (34.33)
Blood in urine	299 (74.93)	100 (25.06)
**In your view, at what age should men start to be more concerned about getting the examination?**		
30 to 40 years	74 (18.54)	
41 to 50 years	203 (50.87)	
Above 51 years	122 (30.57)	
**Effective resources of obtaining information about prostate cancer**		
Health providers	146 (36.59)	
Mass media	112 (28.07)	
Internet	98 (24.56)	
Colleagues	43 (10.77)	

**Table 4:** Logistic regression analysis of variables associated with knowledge of prostate cancer among study participants.

Independent Variables	Groups	B Value	Std. Error	Wald	*p* Value	OR	95% Confidence Interval (CI)
	Intercept	1.604	0.397	16.328	0.000		
Age ranges	Above 60 years	−0.130	0.521	0.063	*p* = 0.803	0.878	0.316–2.439
	51–60 years	0.178	0.370	0.231	*p* = 0.631	1.195	0.578–2.468
	40–50 years	Reference					
Marital status	No	−0.325	0.442	0.540	*p* = 0.462	0.722	0.304–1.719
	Yes						
Personal history of prostate problems	Yes	0.172	0.353	0.238	*p* = 0.626	1.188	0.595–2.371
	No	Reference					
Health insurance	No	−0.139	0.332	0.175	*p* = 0.675	0.870	0.453–1.669
	Yes	Reference					
Level of education	Primary level	0.077	0.908	0.007	*p* = 0.933	1.080	0.182–6.397
	Secondary level	−0.776	0.360	4.635	*p* = 0.031 *	0.460	0.227–0.933
	Other (uneducated)	−0.627	0.572	1.199	*p* = 0.273	0.534	0.174–1.641
	University level	Reference					
Residence	Rural	−0.550	0.387	2.026	*p* = 0.155	0.577	0.270–1.231
	City	Reference					
Occupation	Retired	−0.011	0.397	0.001	*p* = 0.978	0.989	0.454–2.153
	Unemployed	0.852	0.464	3.368	*p* = 0.066	2.345	0.944–5.825
	Employed	Reference					
Family history of prostate problems	Mother’s relative	0.852	0.594	2.057	*p* = 0.152	2.345	0.732–7.519
	From father	0.488	0.323	2.286	*p* = 0.131	1.629	0.865–3.067
	Not known	Reference					
Monthly family income	5000 to 9999 SAR	−0.198	0.389	0.260	*p* = 0.610	0.820	0.383–1.758
	10,000 to 14,999 SAR	−0.197	0.374	0.277	*p* = 0.599	0.821	0.395–1.710
	Above 15,000 SAR	Reference					

In all the independent variables, the last group is the reference category against which the OR is computed. * *p* < 0.05 is considered significant. OR—odds ratio; CI—confidence interval; B value—beta value.

**Table 5:** Logistic regression analysis for knowledge—good [UNADJUSTED MODEL].

Education Level	B	SE	Wald	*p*-Value	OR	95% Confidence Interval (CI)
Primary	–0.182	0.796	0.052	*p* = 0.819	0.833	0.175–3.965
Secondary	–0.742	0.312	5.639	*p* = 0.018 *	0.476	0.258–0.878
Uneducated	–0.385	0.370	1.080	*p* = 0.299	0.681	0.329–1.406
University	Reference	–	–	–	1.000	

* *p* < 0.05 is considered significant. OR—odds ratio; CI—confidence interval; B value—beta value.

**Table 6:** Logistic regression analysis for knowledge—good [ADJUSTED MODEL].

Predictor	B	SE	Wald	*p*-Value	OR	95% Confidence Interval (CI)
Primary	0.028	0.888	0.001	*p* = 0.975	1.028	0.180–5.860
Secondary	–0.732	0.351	4.354	*p* = 0.037 *	0.481	0.242–0.957
Uneducated	–0.693	0.516	1.800	*p* = 0.180	0.500	0.182–1.376
Health insurance (no)	–0.410	0.290	1.995	*p* = 0.158	0.664	0.376–1.172
Residence (rural)	–0.607	0.364	2.787	*p* = 0.095	0.545	0.267–1.111
Retired	–0.016	0.339	0.002	*p* = 0.962	0.984	0.506–1.911
Unemployed	0.945	0.441	4.593	*p* = 0.032 *	2.574	1.084–6.110
Income 5000–9999 SAR	–0.222	0.379	0.342	*p* = 0.559	0.801	0.381–1.684
Income 10,000–14,999 SAR	–0.134	0.363	0.137	*p* = 0.711	0.874	0.429–1.782

* *p* < 0.05 is considered significant. OR—odds ratio; CI—confidence interval; B value—beta value.

**Table 7:** Awareness of prostate cancer screening among study participants.

Awareness of PCa Screening Questions	Category	Number (%)
**Do you know some kinds of examination for cancer detection?**	Digital rectal examination only	149 (37.34)
	Prostate-specific antigen only	74 (18.54)
	DRE and prostate-specific antigen (PSA)	1 (0.25)
	Ultrasound	58 (14.53)
	Do not know	117 (29.32)
**Can early screening increase survival rates?**	Yes	307 (76.94)
	No	92 (23.05)
**Have you ever undergone a prostate-specific antigen (PSA) test?**	Yes	177 (44.36)
	No	197 (49.37)
	Do not remember	145 (36.34)
**When was the last time you underwent a prostate-specific antigen (PSA) test?**	Between one to two years	44 (11.02)
	Less than one year ago	95 (23.80)
	Never	205 (51.37)
	Over three years ago	38 (9.52)
**Awareness regarding treatment**	Drugs/chemotherapy	119 (29.82)
	Surgery	82 (20.55)
	Radiation	62 (15.53)
	Herbal medication	40 (10.02)
	None of the above	96 (24.06)
**Should only men with urinary symptoms be screened?**	Yes	164 (41.10)
	No	186 (46.61)
	Do not know	145 (36.34)
**How important is it to have regular prostate examinations?**	Does not matter	39 (9.77)
	Important	284 (71.17)
	Little or not important at all	76 (19.04)
**Have you ever conducted a prostate exam?**	Yes	156 (39.09)
	No	172 (43.10)
	Do not remember	145 (36.34)
**If so, why was the prostate examination requested?**	Cancer case in the family	26 (16.66)
	Presented symptoms	29 (18.58)
	Prevention	78 (50)
	Participant requested the examination	23 (14.74)

**Table 8:** Logistic regression results for PSA test taken versus sociodemographic variables.

Predictor	Category	B	Std. Error	Wald	*p* Value	OR	95% Confidence Interval (CI)
Intercept		−1.575	0.356	19.583	*p* < 0.001 *		
Age range	Above 60 years	−0.590	0.489	1.453	*p* = 0.228	0.555	0.213–1.447
	51–60 years	0.090	0.335	0.073	*p* = 0.788	1.095	0.567–2.111
	40–50 years	Reference					
Marital status	No	−1.294	0.605	4.571	*p* = 0.033 *	0.274	0.084–0.898
	Yes	Reference					
Personal history of prostate problems	Yes	1.413	0.297	22.656	*p* < 0.001*	4.107	2.296–7.348
	No	Reference					
Health insurance	No	−0.848	0.312	7.374	*p* = 0.007	0.428	0.232–0.790
	Yes	Reference					
Education level	Primary	−0.271	0.784	0.119	*p* = 0.730	0.763	0.164–3.549
	Secondary	−0.043	0.345	0.016	*p* = 0.901	0.958	0.487–1.885
	Other (uneducated)	0.757	0.528	2.055	*p* = 0.152	2.132	0.757–6.005
	University	Reference					
Residence	Rural	0.172	0.385	0.199	*p* = 0.656	1.187	0.558–2.524
	City	Reference					
Occupation	Retired	−0.101	0.364	0.077	*p* = 0.781	0.904	0.442–1.846
	Unemployed	0.588	0.389	2.282	*p* = 0.131	1.800	0.840–3.859
	Employed	Reference					
Family history of prostate problems	Mother’s relative	1.395	0.448	9.688	*p* = 0.002 *	4.033	1.676–9.707
	From father	0.564	0.279	4.081	*p* = 0.043 *	1.758	1.017–3.040
	Not known	Reference					
Monthly family income	5000–9999 SAR	0.260	0.344	0.570	*p* = 0.450	1.297	0.661–2.546
	10,000–14,999 SAR	0.352	0.318	1.223	*p* = 0.269	1.422	0.762–2.652
	Above 15,000 SAR	Reference					

The dependent variable was binary (“Yes” = 1, “No” = 0), and all independent variables were categorical. The reference category for the outcome was “No”. * *p* < 0.05 is considered significant. OR—odds ratio; CI—confidence interval; B value—beta value.

**Table 9:** Logistic regression analysis of variables associated with awareness of prostate cancer screening among study participants.

Independent Variables	Groups	B Value	Std. Error	Wald	*p* Value	OR	95% Confidence Interval (CI)
	Intercept	−0.843	0.331	6.500	*p* = 0.011		
Age ranges	Above 60 years	−0.028	0.467	0.004	*p* = 0.953	0.973	0.389–2.431
	51–60 years	0.232	0.325	0.511	*p* = 0.475	1.262	0.667–2.387
	40–50 years	Reference					
Marital status	No	0.461	0.431	1.147	*p* = 0.284	1.586	0.682–3.688
	Yes	Reference					
Personal history of prostate problems	Yes	1.567	0.288	29.684	*p* < 0.001 *	4.791	2.727–8.418
	No	Reference					
Health insurance	Yes	1.025	0.292	12.334	*p* < 0.001 *	2.786	1.573–4.935
	No	Reference					
Level of education	Primary level	0.071	0.763	0.009	*p* = 0.926	1.073	0.241–4.790
	Secondary level	−0.040	0.333	0.015	*p* = 0.903	0.960	0.501–1.843
	Other (uneducated)	0.483	0.510	0.897	*p* = 0.344	1.621	0.597–4.405
	University level	Reference					
Residence	Rural	−0.459	0.375	1.499	*p* = 0.221	0.632	0.303–1.317
	City	Reference					
Occupation	Retired	−0.589	0.361	2.661	*p* = 0.103	0.555	0.273–1.126
	Unemployed	−0.243	0.376	0.417	*p* = 0.518	0.784	0.375–1.640
	Employed	Reference					
Family history of prostate problems	Mothers relative	0.793	0.437	3.289	*p* = 0.070	2.210	0.938–5.204
	From father	0.504	0.268	3.535	*p* = 0.060	1.656	0.979–2.800
	Not known	Reference					
Monthly family income	5000 to 9999 SAR	0.167	0.326	0.262	*p* = 0.609	1.182	0.624–2.238
	10,000 to 14,999 SAR	0.064	0.308	0.043	*p* = 0.836	1.066	0.582–1.951
	Above 15,000 SAR	Reference					

In all the independent variables, the last group is the reference category against which the OR is computed. * *p* < 0.05 is considered significant. OR—odds ratio; CI—confidence interval; B value—beta value.

**Table 10:** Logistic regression analysis of variables associated with awareness of prostate cancer screening among men without a personal history of prostate problems.

Independent Variables	Groups	B Value	Std. Error	Wald	*p* Value	OR	95% Confidence Interval (CI)
	Intercept	−1.946	1.212	2.579	*p* = 0.108		
Age ranges	40–50 years	0.602	0.846	0.505	*p* = 0.477	1.825	0.347–9.590
	51–60 years	0.275	0.804	0.117	*p* = 0.732	1.317	0.272–6.369
	Above 60 years	Reference					
Marital status	Yes	−0.993	0.510	3.790	*p* = 0.052	0.370	0.136–1.007
	No	Reference					
Health insurance	Yes	0.828	0.410	4.082	*p* = 0.043 *	2.288	1.025–5.109
	No	Reference					
Level of education	Up to secondary level	−0.605	0.892	0.460	*p* = 0.498	0.546	0.095–3.137
	University level	−0.709	0.890	0.635	*p* = 0.426	0.492	0.086–2.818
	Other (uneducated)	Reference					
Residence	Rural	−0.167	0.573	0.085	*p* = 0.771	0.846	0.275–2.602
	City	Reference					
Occupation	Employed	0.774	0.746	1.078	*p* = 0.299	2.169	0.503–9.353
	Unemployed	1.764	0.754	5.476	*p* = 0.019 *	5.834	1.332–25.552
	Retired	Reference					
Family history of prostate problems	Mother’s relative	0.983	0.941	1.091	*p* = 0.296	2.673	0.422–16.917
	From father	0.911	0.436	4.371	*p* = 0.037 *	2.488	1.059–5.847
	Not known	Reference					
Monthly family income	5000 to 9999 SAR	−0.154	0.548	0.078	*p* = 0.779	0.858	0.293–2.511
	10,000 to 14,999 SAR	0.661	0.489	1.828	*p* = 0.176	1.936	0.743–5.048
	Above 15,000 SAR	Reference					

In all the independent variables, the last group is the reference category against which the OR is computed. * *p* < 0.05 is considered significant. OR—odds ratio; CI—confidence interval; B value—beta value. In educational qualification, cases of primary level education were not found for awareness—good category. Due to computational problems, this group was combined with secondary education and renamed as up to secondary level.

**Table 11:** Barriers to screening reported by the study population.

Barriers to Screening	Strongly Disagree Number (%)	Disagree Number (%)	Agree Number (%)	Strongly Agree Number (%)
Unable to afford.	78 (19.54)	92 (23.05)	116 (29.07)	113 (28.32)
In my opinion, I am in good health.	27 (6.76)	95 (23.80)	201 (50.37)	76 (19.04)
I am not sure where to go to get screened.	32 (8.02)	104 (26.06)	171 (42.85)	92 (23.05)
Have no faith that it will stop prostate cancer (PCa).	83 (20.80)	119 (29.82)	108 (27.06)	89 (22.30)
I am worried that during screening they will identify a problem.	52 (13.03)	77 (19.29)	173 (43.35)	97 (24.31)
I am scared it could affect my sexual health.	47 (11.77)	111 (27.81)	161 (40.35)	80 (20.05)
Participant screening is uncomfortable.	58 (14.53)	104 (26.06)	154 (38.59)	83 (20.80)
Think digital rectal exam (DRE) is harmful or embarrassing.	49 (12.28)	117 (29.32)	156 (39.59)	77 (19.29)
Prostate cancer (PCa) screening is time-consuming.	42 (10.52)	119 (29.82)	157 (39.34)	81 (20.30)
Think that PCa is not a serious illness.	80 (20.05)	115 (28.82)	112 (28.07)	92 (23.05)

**Table 12:** ANOVA and *t*-tests for variables versus barriers to prostate cancer screening.

Variables	Groups	Barriers Score			F-Value	t-Value	*p*-Value
		Mean	S.D.	Number			
Age ranges	40–50 years	24.93	6.12	148	11.449	---	*p* < 0.001 *
	51–60 years	28.03	6.29	145			
	Above 60 years	27.42	4.66	106			
Marital status	Yes	26.83	5.97	363	---	1.194	*p* = 0.233
	No	25.58	6.11	36			
Personal history of prostate problems	Yes	28.26	5.94	222	---	6.011	*p* < 0.001 *
	No	24.79	5.48	177			
Health insurance	Yes	27.71	5.98	272	---	4.998	*p* < 0.001 *
	No	24.59	5.43	127			
Level of education	Primary level	26.75	4.11	12	2.608	---	*p* = 0.051
	Secondary level	28	6.32	81			
	University level	26.08	6.07	245			
	Other (uneducated)	27.57	5.18	61			
Residence	Rural	26.84	5.4	117	---	0.254	*p* = 0.800
	City	26.67	6.22	282			
Occupation	Employed	25.83	6.7	180	3.668	---	*p* = 0.026
	Unemployed	27.53	5.37	122			
	Retired	27.34	5.09	97			
Family history of prostate problems	Mothers relative	29.58	7.08	40	17.407	---	*p* < 0.001 *
	From father	27.85	5.11	184			
	Not known	24.88	6.06	175			
Monthly family income	5000 to 9999 SAR	27.51	5.63	130	5.148	---	*p* = 0.006 *
	10,000 to 14,999 SAR	27.16	6.15	153			
	Above 15,000 SAR	25.25	5.94	116			
Have you undergone a prostate specific antigen (PSA) test?	Yes	28.57	6.07	177	---	5.733	*p* < 0.001 *
	No	25.24	5.50	222			

S.D.—standard deviation; * *p* < 0.05 significance; ANOVA—analysis of variance.

## Data Availability

The original contributions presented in the study are included in the article. Further inquiries can be directed to the corresponding author.
